# Medical students as health coaches: adding value for patients and students

**DOI:** 10.1186/s12909-020-02096-3

**Published:** 2020-06-03

**Authors:** Arti Maini, Molly Fyfe, Sonia Kumar

**Affiliations:** Medical Education Innovation and Research Centre (MEdIC), School of Public Health, Imperial College London, Room 331, 3rd floor Reynolds Building, Charing Cross Campus, St Dunstan’s Rd, London, W6 8RP UK

**Keywords:** Health coaching, Patient-centred care, Communication skills

## Abstract

**Background:**

Underlying the global burden of chronic disease are common and modifiable risk factors such as unhealthy diet, physical inactivity and tobacco use. Health coaching is being introduced into healthcare as an effective tool in facilitating behaviour change and addressing lifestyle risk factors in patients. Although some medical schools are training students in health coaching, there is little research on this emerging practice. This qualitative study explores the experience and application of health coaching approaches by third year medical students that have been trained in health coaching.

**Methods:**

Six focus groups were conducted with medical students (*n* = 39) who had participated in an experiential health coaching training module and practiced their health coaching skills in primary care settings. Interactive facilitated discussions between students aimed to explore experiences of health coaching, how this related to their ongoing practice, and their perceived impacts of engagement with patients. Data was thematically analysed.

**Results:**

Themes emerged around ‘mindset’, ‘skills’, ‘application of skills’, ‘perceived value’ and ‘context’. Training in health coaching prompted a shift towards a non-judgemental, solution-oriented mindset in which students increasingly accepted the ability of each person to define their needs and identify individually appropriate solutions. Mindset change supported skill development in person-centred communication, active listening, and self-refection. Mindset and skills related to changes in how students conducted patient consultations, their practice of self-refection, and their personal relationships. Perceived value of coaching approaches reinforced mindset. Students described facilitators to their coaching practice, and also tensions due to misalignment between their coaching mindset and ongoing practices in medical education and service delivery.

**Conclusions:**

Training medical students in health coaching and supporting them to contribute meaningfully through empowering patients in real-world settings can help develop students’ professional identity and a non-judgemental, solution-oriented mindset and skills in self-reflection, person-centred care and facilitating health behaviour change.

## Background

Common and modifiable risk factors such as unhealthy diet, physical inactivity and tobacco use underlie the global burden of chronic disease and multimorbidity in the context of an ageing population [1]. Health coaching has been defined as the art of facilitating a person’s active participation in managing their own health [2]. Coaching is based on the premise that people are resourceful with inner strengths and capabilities, and involves holding non-judgemental, solution-oriented, person-centred conversations to support people to identify and work towards achieving their goals [2]. Health coaching is being introduced into healthcare as an effective approach to addressing lifestyle-related risk factors through behaviour change [3]. There is research to suggest that training healthcare professionals in these skills can support behaviour change in patients, leading to more effective self-care, self-management of long-term conditions, and better health outcomes [4].

There are calls for medical training to address lifestyle management and behaviour change [5], and health promotion is a key theme in the UK’s General Medical Council (GMC) Outcomes for Graduates [6]. To address this, some medical schools are introducing health coaching training into their curricula [7, 8]. Within the medical education literature, health coaching has been identified as a ‘value-added’ approach, through which students can develop professional competence while supporting healthcare delivery [7].

As an emerging educational approach however, there is currently little research on the experience or impacts on medical students involved in health coaching, in particular, how health coaching may contribute to student learning or healthcare delivery. Existing research on health coaching within medical education [7–11] shows that students report high acceptability of health coaching [8, 11] and that students can impact patient outcomes through health coaching in specific contexts [11, 12]. The aim of this study was to explore in depth the experience of medical students trained in health coaching in primary care settings and their perceptions of impacts on their learning and their approaches to patient care.

## Methods

### Context: health coaching in clinical settings

Third-year medical students at Imperial College London undertook an optional health coaching module which consisted of four half-day campus-based small group sessions (of 6–10 students) taking place over four consecutive weeks and embedded within a ten-week community placement in primary care. Health coaching training was designed to integrate small group teaching with opportunities for both supported and independent practice and reflection. At the time of course implementation, this was the only skills-based course in the undergraduate medical school curriculum that focused on lifestyle and behavior change, although the primary care curriculum included a focus on more general consultation skills. The training was developed and delivered by AM who works as a clinician, is a teacher in undergraduate primary care and is accredited as a coach and coach trainer. This combination of experience and expertise enabled development of a bespoke course tailored to the needs and level of medical student participants.

The training was developed in line with principles of health coaching [2] and informed by the conceptual framework of Kolb’s experiential learning theory [13] whereby ‘concrete’ experiences aimed to develop students’ understanding of key principles and skills of health coaching and their ability to apply these in practice with patients. The practice of health coaching does not currently require a formal qualification or licence to practice and this training was not formally accredited. However, from a quality assurance perspective we developed the training in line with generic coaching principles espoused by the European Mentoring and Coaching Council (EMCC) [14]. The purpose of the training was to support students in developing core skills in health promotion and illness prevention [6] through using general health coaching principles and skills to facilitate behaviour change and address lifestyle risk factors in patients. The ethical framework informing health coaching training and practice was based on ethical and professional principles outlined by the GMC [6].

Key skills covered in the training included active listening, reflecting and asking solution-oriented questions to stimulate new thinking, for example in relation to goal-setting, exploration of context and action planning. Training was comprised of: interactive facilitated classroom-based small group discussions and experiential paired and group exercises designed to facilitate exploration of health coaching principles and skills and their application in clinical practice; practicing applying health coaching principles and skills in patient interactions during clinical placements; and facilitated small group reflective discussions of students' experiences in applying these skills with patients. Some exercises involved students working in pairs as both coach and coachee, drawing on their own lifestyle issues as material for coaching practice (‘real play’).

Students were encouraged to use their health coaching skills during their primary care clinical placements, where they had opportunities to consult with patients independently. Students determined when and how they would apply health coaching in their clinical care activities. These activities typically encompassed taking clinical histories during supervised student-run clinics from diverse patients across a wide range of age groups and healthcare needs presenting to primary care, along with more focussed health coaching conversations with patients with long term conditions and modifiable behavioural risk factors who were open to have more in-depth conversations to support health behaviour change.

### Study design

We adopted an exploratory qualitative approach aimed at understanding students’ experience of health coaching and their perceptions of the impact on themselves and their approaches to patient care. We adopted a constructivist stance [15] which poses that knowledge is co-produced through interaction between all involved in the research process. In order to generate rich data which emphasized participant-led interactions, a focus group format was used. Focus groups of 6–8 students and of 45 minutes duration allowed for discussion between students in which they could ask questions of each other and explore experiences among their peer group [16] through interactive exchange.

### Participants and data collection

In total, 225 students participated in the 10-week community placement. Of these, 48 students completed the health coaching course and these students were invited to participate in focus group discussions held shortly after course completion. Thirty-nine students self-selected to participate. Six focus groups were held in 2017–2018, with focus groups being held as each cohort completed the coaching module. Focus groups followed a semi-structured format aimed at facilitating discussion of students’ experience of training, implementation of health coaching approaches and perceptions of impacts. Focus group participants were encouraged to share and compare their experiences and views of implementing coaching skills, and the contexts of their primary care placements (Table 1).

**Table 1:** Questions and prompts used in focus groups

Prompt focus group questions exploring impacts of the training included:	
• How have you used the health coaching approach with patients?	
• How have your patients responded?	
• Have you used this approach any non-clinical situations?	
• Have you noticed any benefits for you personally?	
• What barriers did you experience in using the approach in practice?	
• What factors supported you to use this approach in practice?	
Prompt focus group questions exploring students’ experiences of the training process included:	
• How well do you feel the course prepared you to use coaching approaches with patients?	
• What was the most useful aspect of the course?	
• What was the least useful aspect of the course?	
• What was the most challenging aspect of the course?	
• What was the most surprising aspect of the course?	

### Data analysis

Audio-recordings of focus groups were transcribed verbatim, transcripts were checked for accuracy and all identifying information removed. Data was imported into Dedoose [17] for analysis. We used thematic analysis [18] to allow for inductive (data-driven) themes to be identified by the researchers [19]. We began by immersing ourselves in the data through reading through the focus group transcripts. Researchers (AM and MF) independently reviewed a selection of the data and developed initial codes and code descriptions. We then discussed our initial coding to identify areas of convergence and divergence in our interpretations of the data and reach consensus. Transcripts were next independently coded by each researcher and coding decisions were again reviewed and compared during a series of research meetings until a final coding framework was agreed. Analytic insights were recorded using reflective memos. Codes were grouped into conceptually coherent themes through an iterative process. Focus group data are co-created by researcher and participants, and throughout the research process, we reflected on how our backgrounds and experiences shaped the data collected and our interpretations of it. The research team consisted of a general practitioner, medical educationalist and coach (AM) and an education researcher (MF). Experiential Learning [13] was used as a sensitizing concept in relation to student learning and engagement with health coaching.

## Results

**Fig. 1:**
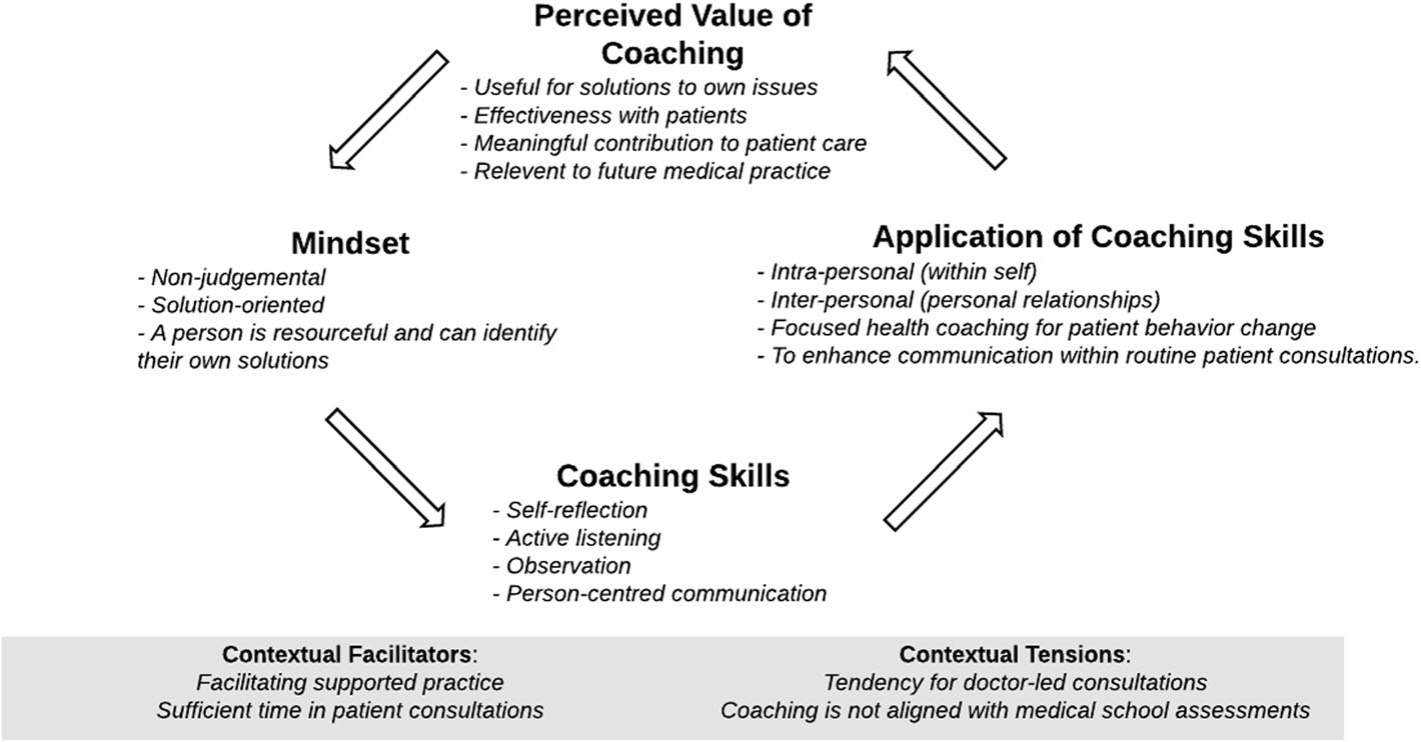
Medical students’ experience of being trained in health coaching: process and impacts

Our analysis of focus groups data resulted in five themes describing the students’ experience of being trained in health coaching: development of a non-judgemental, solution-oriented mindset, coaching skills, application of coaching skills, perceived value of coaching, and facilitators and tensions encountered when introducing health coaching into existing systems (Fig. 1). Students described a shift in mindset generated from experiential learning and described resulting development of coaching skills. Student applied these skills in health coaching conversations, but also more broadly in their interactions with patients and personal relationships. The perception that coaching was an effective approach reinforced mindset, or beliefs.

### Mindset

Students described moving towards a non-judgemental, solution-oriented mindset through developing an increased awareness of the importance of patients defining their own goals and developing their own solutions that took into account their wider life context.

*“Especially with things like lifestyle changes…I think it’s a lot better for them to obviously be able to find the answers to their own problems instead of us just telling them you need lose weight, you need eat better because if that doesn’t fit in with their lifestyle, they’re not going to be able to do it.”*

This corresponded to a shift in their understanding of their own role from ‘giver of advice’ to facilitator of change in another.

*“Now it's more about letting them do the thinking and letting them make the decisions and my role just being the person who helps them in making those decisions but doesn't make it for them.”*

Students repeatedly described the experience of being a ‘coachee’ themselves during training sessions as a catalyst for development of a solution-oriented mindset which assumes that people are resourceful and at some level know the solution to their issues.

*“… that was mind blowing for me … [before doing the exercise, I thought that] this could be a technique that might work but I’m not really sure. But then experiencing it for yourself, that was surprising…I didn’t realise it would actually work so well.”*

*“[It is]really good to see the questions work on yourself… and amazingly you could…change your perception or whatever based on that conversation. But I think it’s really important to be able have it done on you.”*

Students were also able to relate their experience of being coached to what a patient might be experiencing:

*It just makes me feel better that there are things in my life that aren't going right and it's not my fault, and sometimes that helps me. And I feel like that could probably help the patient too who's probably blaming themselves a lot for their problems when actually it's not their fault.*

Students described that based on their changes in mindset towards becoming more non-judgemental and solution-oriented, they adapted their approaches to communication with patients, inter-personal relationships and their own reflective processes. Some students described the change in mindset as enduring.

### Developing and applying coaching skills

Mindset changes were related to students developing and applying skills in person-centred communication, active listening, observation and self-reflection. Learning health coaching skills enabled students to develop communication skills to enable more facilitative conversations relating to person-centred goals, exploring the situation and supporting people to identify options and next steps. They described applying this facilitative approach in a range of clinical and non-clinical situations. Clinical situations included using health coaching for focussed consultations with patients relating to behaviour change as well as using health coaching skills to enhance communication within routine patient consultations. Often students described learning to listen in different ways and placed increased importance on what the patient was saying, becoming more comfortable with use of silence in the process.

*“With normal histories…I get quite uncomfortable with the silence that falls between asking questions…Whereas here, I’ve noticed that I’ve actually got way more comfortable with that silence because you’re giving someone time to think…”*

Students described feeling more prepared when holding conversations with patients related to lifestyle issues (including relating to diet, exercise and smoking cessation). They gave examples of effective shared decision-making and of tailoring conversations to patients’ levels of health literacy when providing lifestyle-related information:

*“It was a patient who was pre-diabetic and the doctor had given information about diet and lifestyle. And when I asked ‘what do you know [already]?’ they said ‘I know I’m not supposed to take sugar, but I don’t take sugar in my coffee or my tea so I don’t see how I’m getting sugar’. I think they felt…the fact that I asked them first, they probably thought all right, she’s not going to just splurge out a speech at me. She actually is going to talk to me about what’s better for me. And then when you did tell them like ‘you know this food has sugar as well’ they’re like ‘oh, I didn’t know that’. And then I think they retain that information, they try and think about that.”*

They also described feeling more confident in holding conversations with patients presenting with multiple issues and with patients presenting with issues which were significantly influenced by psychological and/or social factors in their lives.

Several students commented on how a coaching approach enabled more genuine approaches to asking patients’ questions about their ideas, concerns and expectations (referred to commonly by the acronym ‘ICE’).

*“ICE has become obviously so drilled into us, it feels so fake when we ask it. We are actually concerned and do want to know, but what’s worry you about this, what do you want… It just seems so… And just [coaching] provides another way to do it and another approach to it and it feels more genuine.”*

Medical students applied health coaching skills internally and described changes in their own processes of reflection. Students reported developing resilient approaches through feeling better equipped to think about and identify solutions to their own issues, relating to their lifestyles as well as more generally. Students used coaching approaches successfully at an intrapersonal level, as a strategy for finding solutions to their own problems, resulting in development of resilient thinking processes.

*“I have used it on myself and I think that I normally have a tendency to just keep thinking about things and never really think of a solution, and just keep thinking about what I can't do and what I like…And it's really a bad habit of course but this has literally forced me to think about what I can do and it like completely…changes your perspective.”*

*“I feel more resourceful, I feel like I am more capable of sorting out my own problems instead of going to someone else or just letting them fester”*

### Perceived value of coaching approaches

Students described feeling more confident in clinical consultations through knowing that they could draw on coaching approaches to facilitate constructive conversations without feeling a need to ‘solve’ patients’ problems. They derived a sense of personal satisfaction when able to successfully apply coaching skills to conversations with patients.

*“I think obviously helping people should feel good but I was surprised at how satisfying it can feel to do the coaching, because you’re watching someone do the hard work and then you see them feel good about it, and then it makes you feel… obviously that should make you feel good but it really does…”*

Students perceived that learning and applying a coaching approach was of value to them as it enabled them to not only learn actively rather than being passive observers but also to contribute in a meaningful and rewarding way through impacting positively on patients even whilst at an early stage in their medical training.

*“I felt more empowered as a student … it sounds very cliché, but it gave me more reason to be there because sometimes as a medical student you just feel like you are taking up space …But with this kind of thing then you sort of know there is something you can do.”*

Students perceived relevance of coaching skills to their future work as doctors and several expressed their intention to continue to practice these skills. Students’ perceptions of the value in applying coaching approaches, both for themselves and others, served to reinforce a solution-oriented mindset.

### Context: facilitators and tensions

Factors within the educational and clinical practice settings served to facilitate and limit learning around health coaching. The most prominently described facilitator was that students are often given longer times for consultations with patients, for example twenty minutes rather than the standard ten-minute consultations. Having more time allowed for coaching conversations to occur. Being supported in experiential practice of coaching with opportunities for regular reflection also facilitated learning. Tensions emerged between the person-centred coaching approach students were learning and a different approach they were often witnessing in the GP consultations where the emphasis was on the doctor’s agenda.

*“Often I notice that GPs might have a different agenda to what coaching tries to direct us towards. So whereas we try to explore what the patient thinks is the problem and try to help them reach a decision. The GP might have a goal of say lowering your blood pressure…”*

Coaching approaches, while perceived as valuable, didn’t align with what students felt they were expected to learn for examinations.

*“I think the course is like really valuable but I’m not sure how relevant it is to medical students in particular because a lot of our examinations at least aren’t really on kind of improving health.”*

## Discussion

Our study explored the experience of third year medical students who were trained in health coaching skills, with opportunity to practice these skills in primary care clinical placements. The students consistently described changes in mindset catalysed through reflecting on their experiences of coaching and corresponding to development of more sophisticated communication skills, personal reflexivity, and person-centred attitudes. Students reported transferring the skills and mindset learned in the context of health coaching to other areas of their professional and personal lives. These findings suggest that an experiential approach [13] to health coaching training in which students are supported in linking conceptual development with personal experiences may promote development of important skills for future health professionals: behaviour change, reflexivity, resilience and person-centeredness.

Our findings support previous claims that health coaching is a ‘value-added’ role for medical students [7]. Other studies [11, 12] show how students can impact patient outcomes through health coaching. While our study did not directly measure patient outcomes following health coaching interactions, students did report having meaningful interactions with patients to support their health through using health coaching during focussed consultations with patients about adopting healthier behaviours relating to diet, exercise and smoking cessation, and feeling better equipped to do this. They also felt more confident about approaching consultations more generally, including with patients presenting with multiple issues and with patients presenting with issues which were significantly influenced by psychological and/or social factors in their lives. Students felt better prepared to support shared decision-making processes including tailoring conversations to patients’ levels of health literacy. These areas align with the direction of travel in healthcare interactions and medical education with respect to the desire for greater patient empowerment and recognition of changing patient demographics, with increased prevalence of long term conditions and multimorbidity, and significance of the psychosocial context [1, 6]. Health coaching within the undergraduate medical curriculum may help to more effectively prepare future doctors with the skills necessary to meet the health needs of the population [6]. Further research is needed however to look more directly at patient experiences and outcomes following health coaching interventions with students.

Another study found that taking on a role as health coach motivated medical students to address their own health behaviours [8]. Our study did not directly set out to explore how students’ own lifestyle-related health behaviours were influenced by the health coaching training as the aim of the training was to equip students with skills to apply health coaching approaches to patient interactions rather than to address their own health. However, given that students did report on changes in mindset, it would be valuable for future research to explore the impact of such mindset change on students’ own health behaviours. Students in our study reported developing resilient approaches through developing greater self-awareness and reflective ability and feeling better equipped to think about and identify solutions to their own issues more generally. This has implications for how undergraduate medical curricula may best support students to develop personally and professionally. Students described an increased sense of purpose and value of medical student and doctor roles arising from empowering patients through health coaching approaches, and this may have implications for development of professional identity.

An interesting finding from this study was that once students felt they had developed a solution-oriented mindset, they were then able to see the relevance of asking person-centred questions regarding patients’ ideas, concerns and expectations, and were able to incorporate these more naturally as part of routine patient consultations, something they reported difficulty with prior to the coaching training. Holding a solution-oriented mindset also enabled students to feel more comfortable with silence in conversations, perceiving this as a positive sign that their questions were enabling patients were thinking more deeply about their issues which in turn would contribute to solution-finding. Communication skills previously identified as important to acquire include learning how to elicit patients’ ideas, concerns and expectations, and to appropriately use silence in consultations [20]. The findings of our study therefore have implications for how to more effectively train medical students in communication skills and highlight the value of supporting students to develop a person-centred and solution-oriented mindset at the outset.

Interestingly, some students in this study described influencing change in the practice of other health professionals, suggesting the possibility of a ‘bottom-up’ effect, whereby the health coaching approaches adopted by students had potential to become absorbed into the approaches of their GP tutors. This provides support for the possibility raised in an earlier study [21] that training students in health coaching skills may have a wider impact on the healthcare systems they are placed within.

Students found a number of systemic aspects challenging with respect to implementing health coaching approaches in their GP settings. These included a perceived lack of time and also that a person-centred coaching approach was often not aligned to the approach they were observing during GP consultations which frequently prioritised the doctor’s agenda. It will be important for medical schools and healthcare settings to consider how best to develop learning environments that support students in developing person-centred mindset and skills. A related factor was that while students perceived a person-centred approach to be valuable, they didn’t feel it correlated well with what they would be assessed on in their medical school examinations. A key driver for student learning relates to the assessment process [22], and medical schools are increasingly considering how best to align assessment with the need for students to develop person-centred approaches. Many participants in this study had self-selected to train in health coaching skills and views expressed may reflect pre-existing interests in coaching approaches. Further research to investigate health coaching within a broader cohort of students and to explore longer term impacts of health coaching is suggested.

### Limitations

Several issues should be taken into account when interpreting the findings from this study. Firstly, most, though not all, students participating in the health coaching training had self-selected to undertake the coaching module and students further self-selected to participate in the study focus groups. Therefore, study findings most likely represent the views of students with existing interest in health coaching.

One of the authors of this study, AM, was involved with training students in coaching skills as well as with data analysis. AM’s perspective as a clinician, coach and coach trainer contributed to rich discussion and insights into the data, however involvement as both the course lead and researcher may introduce assumptions or confirmation bias into qualitative data interpretation.

Our study did not directly set out to explore how students’ own lifestyle-related health behaviours were influenced by the health coaching training. We also did not explore directly the changes that patients were making relating to their own health habits. We suggest these as areas for future work.

## Conclusion

Health coaching approaches are increasingly being used within healthcare contexts as a way of addressing behaviour change, and they are being introduced as a skill for medical students to develop and draw upon during their interactions with patients. The findings of this study demonstrate that experiential learning of health coaching approaches, including students themselves being recipients of health coaching, can facilitate development of a solution-oriented mindset and significant learning in multiple domains, including communication, behaviour change, personal reflexivity, resilience and person-centredness. The findings also demonstrate how this mindset shift is reinforced by students being placed in real-world experiential situations with opportunity to empower patients through applying health coaching skills during patient interactions. Students can gain satisfaction and a sense of purpose through contributing meaningfully at an early stage in their medical training. These findings have implications for development of person-centred approaches and of professional identity in medical students and highlight a need for medical schools and healthcare settings to develop learning environments that support students in developing these further.

## Data Availability

The datasets used and/or analysed during the current study are available from the corresponding author on reasonable request.

## Abbreviations

GMC: General Medical Council
EMCC: European Mentoring and Coaching Council
ICE: Ideas, Concerns and Expectations
GP: General Practice

## References

[1] World Health Organization. Global action plan for the prevention and control of noncommunicable diseases 2013–2020. Geneva: WHO Press: 2013.

[2] Rogers J, Maini A. Coaching for health: why it works and how to do it. Maidenhead: Open University Press; 2016.

[3] Kivelä K, Elo S, Kyngäs H, Kääriäinen M (2014). The effects of health coaching on adult patients with chronic diseases: a systematic review. Patient Educ Couns.

[4] The Evidence Centre for Health Education East of England. Does health coaching work? Summary of key themes from a rapid review of empirical evidence. Health Education East England: NHS. Available from: https://eoeleadership.hee.nhs.uk/sites/default/files/Does%20health%20coaching%20work%20-%20summary.pdf Accessed 15 July 2016.

[5] Hivert MF, Arena R, Forman DE, Kris-Etherton PM, McBride PE, Pate RR, Spring B, Trilk J, Van Horn LV, Kraus WE (2016). Medical training to achieve competency in lifestyle counseling: an essential foundation for prevention and treatment of cardiovascular diseases and other chronic medical conditions: a scientific statement from the American Heart Association. Circulation..

[6] General Medical Council (2018). Outcomes for Graduates 2018.

[7] Gonzalo JD, Dekhtyar M, Hawkins RE, Wolpaw DR (2017). How can medical students add value? Identifying roles, barriers, and strategies to advance the value of undergraduate medical education to patient care and the health system. Acad Med.

[8] Polak R, Finkelstein A, Axelrod T, Dacey M, Cohen M, Muscato D, Shariv A, Constantini NW, Brezis M (2017). Medical students as health coaches: implementation of a student-initiated lifestyle medicine curriculum. Isr J Health Policy Res.

[9] Wagner PJ, Jester DM, Moseley GC (2002). Medical students as health coaches. Acad Med.

[10] Ahluwalia S, de Silva D, Kumar S, Viney R, Chana N (2013). Teaching GP trainees to use health coaching in consultations with patients: evaluation of a pilot study. Educ Prim Care.

[11] Krok-Schoen JL, Shim R, Nagel R, Lehman J, Myers M, Lucey C, Post DM (2017). Outcomes of a health coaching intervention delivered by medical students for older adults with uncontrolled type 2 diabetes. Gerontol Geriatr Educ.

[12] Kaplan JA, Brinson Z, Hofer R, O'Sullivan P, Chang A, Horvath H, Chang GJ, Finlayson E (2017). Early learners as health coaches for older adults preparing for surgery. J Surg Res.

[13] Kolb DA (1984). Experiential learning: experience as the source of learning and development.

[14] European Mentoring and Coaching Council. EMCC Competence Framework V2 September 2015. European Mentoring and Coaching Council. Available from: https://www.emccouncil.org/wp-content/uploads/2018/10/EMCC-competencesframework-v2-EN.pdf Accessed 12 July 2016.

[15] Schwandt TA (1994). Constructivist, interpretivist approaches to human inquiry.

[16] Stalmeijer RE, McNaughton N, Van Mook WN (2014). Using focus groups in medical education research: AMEE Guide No. 91. Med Teach.

[17] SocioCultural Research Consultants. Dedoose Version 8.0.35, web application for managing, analyzing, and presenting qualitative and mixed method research data [Internet]. Available from: https://www.dedoose.com. Accessed 13 Nov 2018.

[18] Braun V, Clarke V (2006). Using thematic analysis in psychology. Qual Res Psychol.

[19] Varpio L, Ajjawi R, Monrouxe LV, O'Brien BC, Rees CE (2017). Shedding the cobra effect: problematising thematic emergence, triangulation, saturation and member checking. Med Educ.

[20] Kurtz S, Draper J, Silverman J. Teaching and learning communication skills in medicine. Abingdon: Radcliffe Publishing Ltd; 2005.

[21] Leedham-Green K, Wylie A, Ageridou A, Knight A (2019). Brief intervention for obesity in primary care: how does student learning translate to the clinical context?. MedEdPublish.

[22] Buss B, Krautter M, Möltner A, Weyrich P, Werner A, Jünger J, Nikendei C (2012). Can the ‘assessment drives learning’ effect be detected in clinical skills training? Implications for curriculum design and resource planning. GMS Z Med Ausbild.

